# The Nonlinear Dynamics of a MEMS Resonator with a Triangular Tuning Comb

**DOI:** 10.3390/mi14112109

**Published:** 2023-11-17

**Authors:** Lijuan Zhang, Huabiao Zhang, Xinye Li, Ningguo Qiao, Xianping Gao, Yunxiao Ji

**Affiliations:** 1 School of Automobile and Transportation, Tianjin University of Technology and Education, Tianjin 300222, China; qiaoningguo@163.com (N.Q.); gaoxianping1203@163.com (X.G.); 2 School of Mechanical Engineering, Tianjin University of Commerce, Tianjin 300134, China; hbzhang@tjcu.edu.cn; 3 School of Mechanical Engineering, Hebei University of Technology, Tianjin 300401, China; xylihebut@163.com; 4 Tianjin Juxinhongtai Metal Material Company Ltd., Tianjin 300112, China; yunxiaojijxht@163.com

**Keywords:** nonlinear dynamics, MEMS resonator, triangular tuning comb, periodic solution, dis-smooth tuning force

## Abstract

The nonlinear dynamic response of a MEMS resonator with a triangular tuning comb is studied. The motion equation with dis-smooth tuning electrostatic force is derived according to Newton’s second law. The analytical solution of the periodic response is obtained using the harmonic balance method and section integral method. The singularity theory is then applied to investigate the bifurcation of the periodic response of the untuned system. The transition sets on the DC-AC voltage plane dividing the planes into several persistent regions are obtained. The bifurcation diagrams’ topological structures and jump phenomena corresponding to different parameter regions are analyzed. We explore the effects of tuning voltage on the response. This demonstrates that the amplitude–frequency curves present more hardening characteristics with increased tuning voltage. Many twists, bifurcation points, and unstable solutions appear, leading to complicated jump phenomena. Two bifurcation points exist on the response curves: the smooth and dis-smooth bifurcation points, with the latter occurring on the switching plane of non-uniform fingers.

## 1. Introduction

MEMS comb resonators are widely used in signal processing, gyroscopes, oscillators, etc., due to their advantages in terms of signal-to-noise ratio, quality factor, and sensitivity [1]. Most MEMS comb resonators typically feature fingers of equal cross-section and length. Recently, researchers have explored alternatives, including combs with non-uniform finger lengths which can produce a displacement-dependent electrostatic force. This has gained significant attention due to its potential applications in modifying the stiffness and resonance frequency of resonators.

Lee et al. [2] first presented a triangular comb array of linearly varied finger lengths for frequency tuning, and reduced the resonant frequency by changing the tuning voltage. Dai et al. [3] investigated the fabrication of a micromechanical tunable resonator. The tuning unit has a movable comb with constant finger length and a fixed comb with linearly varied finger length. Kao et al. [4] presented the design and fabrication of a micromechanical tunable in-plane resonator with non-uniform finger lengths. The effects of the geometric shape of the tuning part and the tuning voltage-to-stiffness ratio on the tuned frequency were discussed. Shmulevich et al. [5,6] believed that the comb drives with tapered fingers produce an electrostatic force proportional to the applied voltage and the motion. They used the device as an electrostatic anti-spring for frequency tuning of the first instability window of a MEMS Meissner parametric resonator. Kavitha et al. [7] presented a design of a MEMS accelerometer with varied finger lengths for sensing low-frequency low-amplitude accelerations. Taherian et al. [1,8] proposed a MEMS comb tunable resonator featuring a triangular comb with non-uniform finger lengths to expand the tuning resonant frequency range while minimizing the required tuning voltage. These studies have been conducted on the design and fabrication of a resonator with combs with variable finger lengths and the variability of the tuned frequency. However, few studies have focused on the equally important aspect of the resonator’s response.

The response of the MEMS resonator can be greatly affected by nonlinearity factors. The nonlinearity of the suspension and electrostatic force may result in specific response characteristics, such as hardening/softening/mixture behaviors and pull-in. These characteristics could eventually impact the resonator’s performance [9]. Therefore, much work has been done on the nonlinear dynamics of the MEMS comb resonator. Nguyen et al. [10] reported a monolithic comb drive oscillator with high-Q and modeled its operation and performance. Jeong et al. [11] showed a theoretical method to obtain the maximum linear displacement of the actuating comb using a nonlinear dynamic model. Zhang et al. [12] studied a MEMS resonator’s nonlinear responses and dynamics under two-frequency parametric and external excitations. The effect of varying the DC bias, the squeeze film damping, cubic stiffness, and AC excitation amplitude on the frequency response curves, resonant frequencies, and nonlinear dynamic characteristics, were given. Elshurafa et al. [13] discussed the softening and hardening phenomena of a resonator. They derived the electrostatic force considering both the transverse and longitudinal capacitances of the combs and gave the linear and nonlinear spring constants of the support beams by solving a nonlinear boundary value problem. Tusset et al. [14] studied the chaotic behaviors of the MEMS resonator in a fractional order by historical time and phase portraits. Khirallah [15] added a new electrode that generates an electrostatic force on the truss for the parametric excitation, parametric amplification, and linear and nonlinear tuning of a folded-beam comb drive oscillator. Zhong et al. [16] considered the effects of the inclination of the comb fingers caused by fabrication error. Han et al. [17] investigated the primary resonance of a folded-MEMS comb drive resonator and presented a zoning diagram depicting the extreme vibration amplitude versus DC voltage regarding the hardening and softening bending of response branches and the ideal estimation of dynamic pull-in instability. Zhang et al. [9] investigated the bifurcation characteristics of a resonator and gave the transition sets of different bifurcation behaviors on the parameter plane. The jump phenomena and the influences of the parameters on the amplitude–frequency response were discussed. By disconnecting the rotor and keeping it electrostatically floating, Kassie et al. [18] turned the common resonator into a parametric resonator and gave theoretical and experimental explanations for the considerably large response. Ramanan et al. [19] derived a nonlinear dynamic model with a damping constant obtained from a Monte Carlo simulation and described the vibration responses of micro-resonators operating in the nonlinear region.

In these investigations, researchers typically considered the cubic nonlinear restoring force of the suspension structure and the fractional electrostatic force. However, in resonators with non-uniform finger lengths, there is an additional dis-smooth component of the electrostatic force. Previous studies on the nonlinear dynamics of MEMS resonators have not fully considered this dis-smooth electrostatic force despite its presence in variable-length comb resonators. In refs. [2,3,4,5,6,7,8], the dis-smooth electrostatic force is simplified to a linearly varying force with displacement, which can lead to solution errors. Therefore, it is essential to study the nonlinear dynamics of variable-length comb resonators while taking into account the influence of dis-smooth electrostatic forces.

This study examines the nonlinear dynamics of a MEMS resonator that contains a triangular tuning comb. The innovation lies in considering the dis-smooth electrostatic force of the comb with non-uniform finger lengths and the analytical solution of periodic response based on the harmonic balance method and section integration. We also investigate the impact of parameters on response bifurcation. The results provide a theoretical foundation for designing and utilizing MEMS resonators with non-uniform finger lengths.

The paper’s structure is as follows: In Section 2, the dynamic equations of the MEMS resonator are established based on Newton’s second law, and the electrostatic force of the comb with non-uniform finger lengths is derived. In Section 3, the analytical solution of the periodic response is obtained using the harmonic balance method and section integral method. The stability of the periodic solution is analyzed. In Section 4, the effects of the DC and AC voltages on the bifurcation behaviors of the untuned system are discussed using the singularity theory. The dynamic response of the tuning system is analyzed in Section 5. Finally, the conclusion is summarized.

## 2. Physical Model of the Resonator and Its Mathematical Description

**Figure 1:**
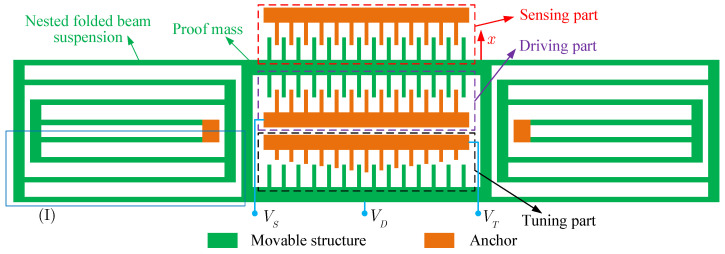
Schematic diagram of MEMS resonator with a triangular tuning comb [1].

We consider a tunable MEMS resonator, as shown in Figure 1 [1]. A nested folded beam suspension supports the proof mass of the resonator. The resonator has a driving part, a sensing part, and a tuning part. The driving and sensing parts consist of movable, fixed combs with constant finger lengths. The tuning comb contains a movable comb of constant finger length and a fixed comb of varied finger length. The DC voltage $V_{D}$ is applied to the movable structure, the AC voltage $V_{S}=V_{A}cos\omega t$ is applied to the fixed driving comb, and the proof mass is actuated under the action of the electrostatic force generated by the voltages. The sensing part generates variation in capacitance during the operation of the resonator. When applying a voltage $V_{T}$ to the fixed tuning comb, the electrostatic force can tune the resonant frequency and the response.

**Figure 2:**
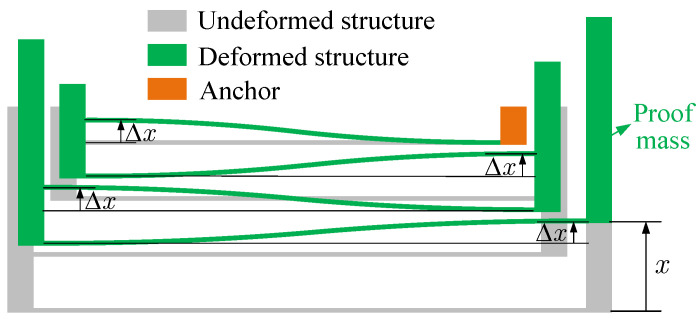
Local enlargement of (I) in Figure 1 and the deformation of the suspension structure when the proof mass has a displacement *x*.

According to Newton’s second law, the motion equation of the resonator can be given as follows:

(1) $$m\ddot{x}+c\dot{x}+k_{1}x+k_{3}x^{3}=F_{t}+F_{d}+F_{s}$$

where *m* is the proof mass, $k_{1}$ and $k_{3}$ are the linear and cubic nonlinear stiffness coefficients, and $F_{d},F_{s}$, and $F_{t}$ denote the driving, sensing, and tuning electrostatic force, respectively. Figure 2 shows the local enlargement of (I) in Figure 1 and the deformation of the suspension structure. Assuming that the length of the supporting beams is equal, when the proof mass has a displacement *x*, the deformation of each supporting beam is

(2) Δx=x/4

According to ref. [13], the restoring force of the structure in Figure 2 can be obtained as follows:

(3) $${\tilde{F}}_{re}=\frac{12EI}{L^{3}}\Delta x+\frac{432EI}{35L^{5}}\Delta x^{3}=\frac{3EI}{L^{3}}x+\frac{27EI}{140L^{5}}x^{3}$$

where *E* is the Young’s modulus of the resonator, $I=hb^{3}/12$ is the section modulus of the supporting beams, *h* is the structural thickness, and *b* and *L* are the width and length of the beams. Then, the restoring force of the suspension of the resonator is

(4) $$F_{re}=4{\tilde{F}}_{re}=k_{1}x+k_{3}x^{3}=\frac{12EI}{L^{3}}x+\frac{27EI}{35L^{5}}x^{3}$$

**Figure 3:**
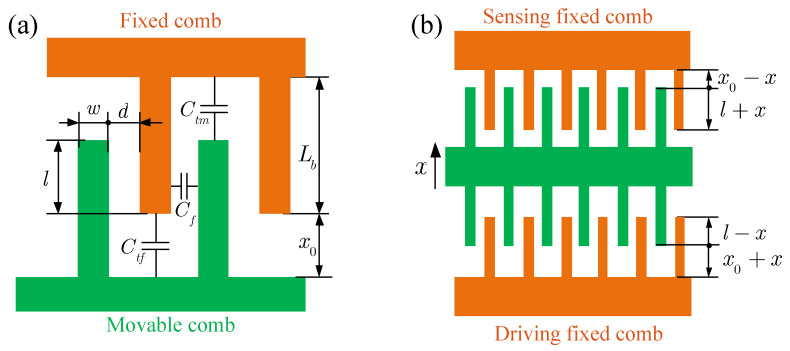
The schematic diagram of the structure, capacitance, and operation of the combs with uniform finger length. (**a**) The structure and capacitance of the combs; (**b**) the operation of the driving and sensing combs.

To obtain the expression of the electrostatic forces, the capacitance of the combs should be analyzed. The comb capacitance consists of three parts (see Figure 3a): the capacitance between the fixed finger and the movable finger $C_{f}$, the capacitance at the tip of the fixed finger $C_{tf}$, and the capacitance at the tip of the movable finger $C_{tm}$. For the combs with uniform finger length [13]

(5) $$C_{f}=\varepsilon la_{1},C_{tf}=C_{tm}=\frac{\varepsilon hw}{x_{0}}$$

where

(6) $$a_{1}=\frac{h}{d}+\frac{1}{\pi }\ln\left(\left({\left(1+\frac{w}{d}\right)}^{2}-1\right){\left(\;\frac{2d}{w}+1\right)}^{1+\frac{w}{d}}\right)$$

*w* is the finger width, *d* is the spacing between the fingers, and $x_{0}$ and *l* are the static separation and overlap between the fixed and moving combs. ϵ is the dielectric constant.

When the proof mass has a displacement *x* (shown in Figure 3b), the separation and the overlaps between the combs are changed, and the driving and sensing capacitance are

(7) $$\begin{array}{c} C_{d}=2\varepsilon N\left(l-x\right)a_{1}+\;\frac{2\varepsilon Nhw}{x_{0}+x},\\ C_{s}=2\varepsilon N\left(l+x\right)a_{1}+\frac{2\varepsilon Nhw}{x_{0}-x} \end{array}$$

where *N* is the number of fingers on a single side of the driving and sensing combs; thus, the electrostatic forces in the driving and sensing directions can be expressed as

(8) $$\begin{array}{c} F_{d}=\frac{1}{2}{\left(V_{S}-V_{D}\right)}^{2}\frac{\partial C_{d}}{\partial x}=-\varepsilon N{\left(V_{S}-V_{D}\right)}^{2}\left(a_{1}+\frac{hw}{{\left(x_{0}+x\right)}^{2}}\right),\\ F_{s}=\frac{1}{2}{V_{D}}^{2}\frac{\partial C_{s}}{\partial x}=\varepsilon N{V_{D}}^{2}\left(a_{1}+\frac{hw}{{\left(x_{0}-x\right)}^{2}}\right) \end{array}$$

**Figure 4:**
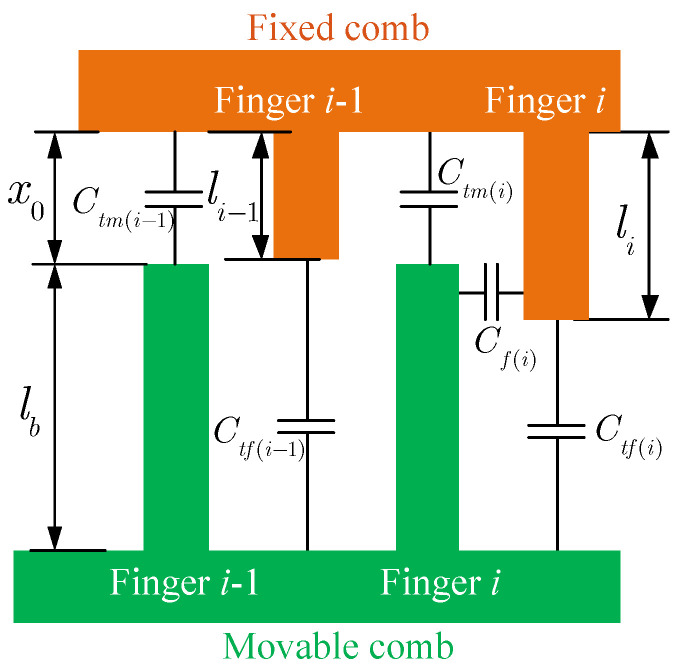
The schematic diagram of the structure and capacitance of the combs with varied finger lengths.

The movable fingers of the tuning comb are uniform (see Figure 4). The tip capacitance of the *i*th movable finger is

(9) $$C_{tm(i)}=\frac{\varepsilon hw}{x_{0}}$$

The length of the fixed fingers is non-uniform, and the separation of each finger is different. Thus, the tip capacitance of the *i*th fixed finger is

(10) $$C_{tf(i)}=\frac{\varepsilon hw}{x_{0(i)}}$$

where $x_{0(i)}=L-l_{i}$, *L* is the static separation between fixed and movable electrodes, and $l_{i}$ denotes the length of the *i*th finger of the fixed comb. The capacitance between the fixed and movable fingers depends on the overlap. There is a capacitance when the movable and fixed fingers overlap (see finger *i* in Figure 4). The capacitance should be zero if the fingers do not overlap (see finger i−1 in Figure 4). Thus, the capacitance between *i*th movable and fixed fingers can be given as

(11) $$C_{f(i)}=\delta \left(l_{i}-x_{0}\right)a_{1}$$

where

(12) $$\delta =\left\{\begin{array}{cc} 1 & l_{i}-x_{0}>0,\\ 0 & l_{i}-x_{0}\leq 0 \end{array}\right.$$

Therefore, the tuning capacitance as the proof mass has a displacement *x* is

(13) $$C_{t}=\;\sum_{i=1}^{N}2\varepsilon \delta \left(l_{i}-x_{0}+x\right)a_{1}+\sum_{i=1}^{N}\left(\frac{\varepsilon hw}{L-l_{i}-x}\right)+\frac{\varepsilon Nhw}{x_{0}-x}$$

where

(14) $$\delta =\left\{\begin{array}{cc} 1 & l_{i}-x_{0}+x>0,\\ 0 & l_{i}-x_{0}+x\leq 0 \end{array}\right.$$

Then, the tuning electrostatic force is obtained as follows:

(15) $$\begin{array}{c} F_{t}=\frac{1}{2}{\left(V_{D}-V_{T}\right)}^{2}\frac{\partial (C_{tl}+C_{tt})}{\partial x}\\ ={\left(V_{D}-V_{T}\right)}^{2}\left(\sum_{i=1}^{N}\varepsilon \delta a_{1}+\sum_{i=1}^{N}\frac{\varepsilon hw}{2{\left(L-l_{i}-x\right)}^{2}}+\frac{\varepsilon Nhw}{2{\left(x_{0}-x\right)}^{2}}\right) \end{array}$$

**Figure 5:**
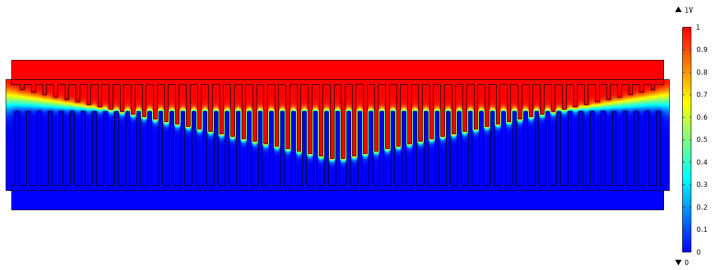
The electric potential distribution of the tuning electrode, where $V_{D}=0,V_{T}=1\;V,$ x=10μm.

**Figure 6:**
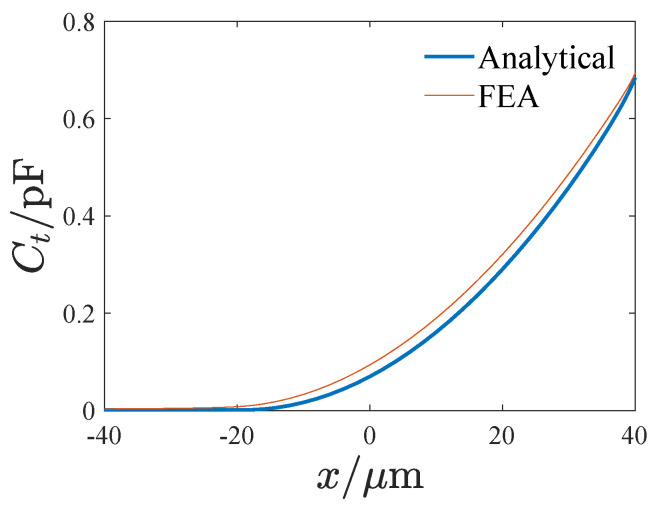
The effect of the displacement on the tuning capacitance, where $C_{t}$ denotes the tuning capacitance and *x* is the displacement.

**Figure 7:**
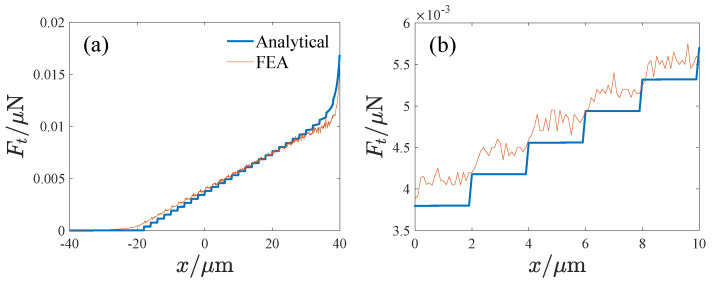
The effect of the displacement on the tuning capacitance, where (**b**) is the local enlargement of (**a**), $F_{t}$ denotes the tuning electrostatic force, *x* is the displacement, and $V_{D}=0,V_{T}=1\;V$.

Note that $l_{i}-x_{0}+x=0$ is a critical state, and when *x* crosses the critical state, $F_{t}$ will change abruptly. Finite element analysis is performed to verify the tuning electrostatic force. Figure 5 presents the electric potential distribution of the tuning electrode. Figure 6 shows the influence of the displacement on the tuning capacitance, where $C_{t}$ denotes the tuning capacitance and *x* is the displacement. It can be seen that the theoretical result is approximate to the FEA result. The difference may stem from the insufficient consideration of the edge field in the theoretical result. Figure 7 gives the effect of the displacement on the tuning electrostatic force, where the electrostatic force $F_{t}$ is calculated using the differential of the tuning capacitance so that the FEA result appears to be fluctuating, but the sudden jumps in the force are clearly visible.

By setting $x=Xx_{0},\tau =\omega t$, the motion equation can be written into the non-dimensional form as

(16) $$X^{\prime \prime }+\zeta X^{\prime }+\Omega^{2}X+\alpha X^{3}=F_{T}+F_{D}+F_{S}$$

where

(17) $$\begin{array}{c} F_{D}=C{\left(\beta \cos\tau -1\right)}^{2}\left(-a_{1}-\frac{a_{2}}{{\left(1+X\right)}^{2}}\right),F_{S}=C\left(a_{1}+\frac{a_{2}}{{\left(1-X\right)}^{2}}\right),\;\\ F_{T}=c_{4}a_{1}f\left(\delta \right)+\frac{1}{2}c_{4}a_{4}\sum_{i=1}^{N}\frac{1}{{\left(L_{i}-X\right)}^{2}}+\frac{1}{2}Nc_{4}a_{2}\frac{1}{{\left(1-X\right)}^{2}},\;\\ \omega_{0}=\sqrt{k/m},\zeta =\frac{c}{m\omega },\alpha =\frac{k_{3}{x_{0}}^{2}}{m\omega^{2}},\Omega^{2}=\frac{k_{1}}{m\omega^{2}},\beta =\frac{V_{A}}{V_{D}},\;\\ a_{2}=\frac{hw}{{x_{0}}^{2}},C=\frac{\varepsilon N{V_{D}}^{2}}{m\omega^{2}x_{0}},c_{4}=\frac{\varepsilon {\left(V_{D}-V_{T}\right)}^{2}}{mx_{0}\omega^{2}} \end{array}$$

where

(18) $$\begin{array}{c} f\left(\delta \right)=\sum_{i=1}^{N}\delta ,\delta =\left\{\begin{array}{cc} 1 & L_{ti}+X>0,\\ 0 & L_{ti}+X\leq 0, \end{array}\right.\\ L_{i}=\left(L-l_{i}\right)/x_{0}\\ L_{ti}=\left(l_{i}-x_{0}\right)/x_{0} \end{array}$$

**Figure 8:**
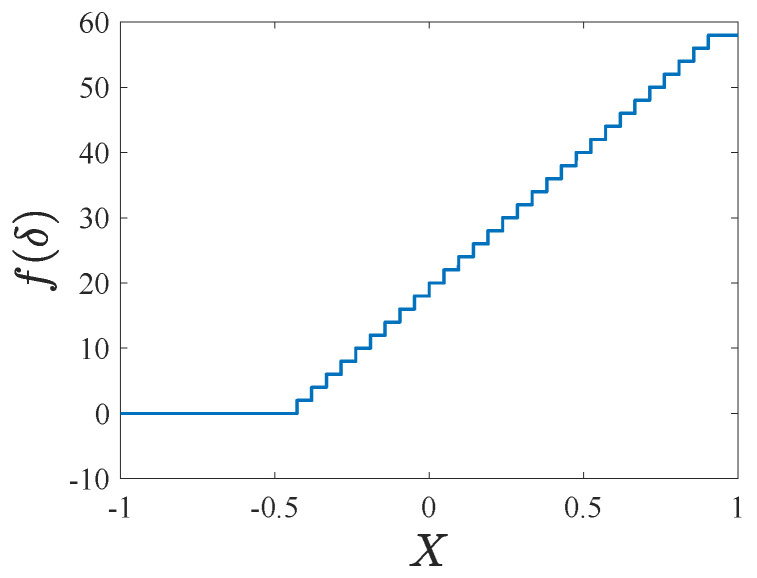
The curve of f(δ) versus *X*.

In our study, the fixed tuning comb is set to be triangular. For the given data in Table 1, f(δ) can be rewritten as

(19) $$f\left(\delta \right)=\left\{\begin{array}{cc} 0 & X\leq -\frac{9}{21}\\ 18+2j & -\frac{9}{21}\leq \frac{j-1}{21}<X\leq \frac{j}{21}\leq \frac{19}{21}\\ 58 & X>\frac{19}{21} \end{array}\right.$$

where *j* is an integer. Figure 8 shows the f(δ) curve versus the non-dimensional displacement *X*. Notice that f(δ) is dis-smooth, and the switching plane of f(δ) is j/21.

## 3. Analytical Solution of the Periodic Response and Its Stability

Periodic response is essential to the performance of resonators. In this section, the harmonic balance method is used to calculate the periodic response of the system. Considering that tuning may cause the equilibrium position of the response to deviate from 0, the solution is assumed as

(20) $$X=A_{0}+A_{1}\cos\tau =A_{0}+A_{1}\cos\left(\tau +\theta \right)$$

By substituting Equation (20) into Equation (16), and extracting the coefficients of cosτ,sinτ, and the constant term through Fourier expansion, the equations about the amplitudes $A_{0},A_{1}$ and phase angular θ can be given as [20]

(21) $$\begin{array}{c} f_{0}=\frac{1}{2\pi }\int_{0}^{2\pi }X^{\prime \prime }+\zeta X^{\prime }+\Omega^{2}X+\alpha X^{3}-\left(F_{T}+F_{D}+F_{S}\right)d\tau \\ =\frac{1}{2\pi }\underset{F_{0}}{\underset{︸}{\int_{0}^{2\pi }X^{\prime \prime }+\zeta X^{\prime }+\Omega^{2}X+\alpha X^{3}d\tau }}-\frac{1}{2\pi }\underset{F_{D0}}{\underset{︸}{\int_{0}^{2\pi }F_{D}d\tau }}-\frac{1}{2\pi }\underset{F_{S0}}{\underset{︸}{\int_{0}^{2\pi }F_{S}d\tau }}\\ -\frac{c_{4}a_{4}}{4\pi }\sum_{i=1}^{N}\underset{F_{T01}}{\underset{︸}{\int_{0}^{2\pi }\frac{1}{{\left(L_{i}-X\right)}^{2}}d\tau }}-\frac{Nc_{4}a_{4}}{4\pi }\underset{F_{T02}}{\underset{︸}{\int_{0}^{2\pi }\frac{1}{{\left(1-X\right)}^{2}}d\tau }}-\frac{c_{4}a_{3}}{2\pi }\underset{F_{T0}}{\underset{︸}{\int_{0}^{2\pi }f(\delta )d\tau }} \end{array}$$

(22) $$\begin{array}{c} f_{1}=\frac{1}{\pi }\int_{0}^{2\pi }[X^{\prime \prime }+\zeta X^{\prime }+\Omega^{2}X+\alpha X^{3}-\left(F_{T}+F_{D}+F_{S}\right)]\cos\tau d\tau \\ =\frac{1}{\pi }\underset{F_{1}}{\underset{︸}{\int_{0}^{2\pi }(X^{\prime \prime }+\zeta X^{\prime }+\Omega^{2}X+\alpha X^{3})\cos\tau d\tau }}-\frac{1}{\pi }\underset{F_{D1}}{\underset{︸}{\int_{0}^{2\pi }F_{D}\cos\tau d\tau }}-\frac{1}{\pi }\underset{F_{S1}}{\underset{︸}{\int_{0}^{2\pi }F_{S}\cos\tau d\tau }}\\ -\frac{c_{4}a_{4}}{2\pi }\sum_{i=1}^{N}\underset{F_{T11}}{\underset{︸}{\int_{0}^{2\pi }\frac{1}{{\left(L_{i}-X\right)}^{2}}\cos\tau d\tau }}-\frac{Nc_{4}a_{4}}{2\pi }\underset{F_{T12}}{\underset{︸}{\int_{0}^{2\pi }\frac{1}{{\left(1-X\right)}^{2}}\cos\tau d\tau }}-\frac{c_{4}a_{3}}{\pi }\underset{F_{T1}}{\underset{︸}{\int_{0}^{2\pi }f(\delta )\cos\tau d\tau }} \end{array}$$

(23) $$\begin{array}{c} f_{2}=\frac{1}{\pi }\int_{0}^{2\pi }[X^{\prime \prime }+\zeta X^{\prime }+\Omega^{2}X+\alpha X^{3}-\left(F_{T}+F_{D}+F_{S}\right)]\sin\tau d\tau \\ =\frac{1}{\pi }\underset{F_{2}}{\underset{︸}{\int_{0}^{2\pi }(X^{\prime \prime }+\zeta X^{\prime }+\Omega^{2}X+\alpha X^{3})\sin\tau d\tau }}-\frac{1}{\pi }\underset{F_{D2}}{\underset{︸}{\int_{0}^{2\pi }F_{D}\sin\tau d\tau }}-\frac{1}{\pi }\underset{F_{S2}}{\underset{︸}{\int_{0}^{2\pi }F_{S}\sin\tau d\tau }}\\ -\frac{c_{4}a_{4}}{2\pi }\sum_{i=1}^{N}\underset{F_{T21}}{\underset{︸}{\int_{0}^{2\pi }\frac{1}{{\left(L_{i}-X\right)}^{2}}\sin\phi \tau d\tau }}-\frac{Nc_{4}a_{4}}{2\pi }\underset{F_{T22}}{\underset{︸}{\int_{0}^{2\pi }\frac{1}{{\left(1-X\right)}^{2}}\sin\tau d\tau }}-\frac{c_{4}a_{3}}{\pi }\underset{F_{T2}}{\underset{︸}{\int_{0}^{2\pi }f(\delta )\sin\tau d\tau }} \end{array}$$

Except for $F_{T0},F_{T1},F_{T2}$, other terms can be obtained through the integral of residue theorem [21]. The detailed calculation process and results are shown in Appendix A.

**Figure 9:**
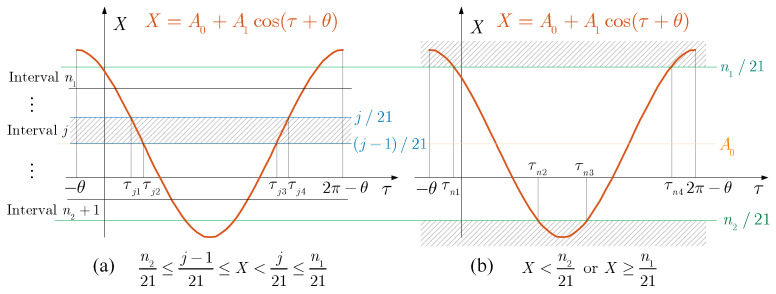
The schematic diagram of the integral of f(δ).

Figure 9 shows the schematic diagram of the integral of f(δ). Considering f(δ) is non-smooth, its integral must be carried out in sections equal to each section’s sum. Since $F_{T}\geq 0$, $A_{0}\geq 0$, the maximum value for displacement *X* is $A_{0}+A_{1}$, and the minimum value is $A_{0}-A_{1}$. According to the expression of f(δ), we set

(24) $$\begin{array}{c} n_{1}=\left\{\begin{array}{cc} \left\lfloor 21(A_{0}+A_{1})\right\rfloor & \mathrm{else}\\ 19 & n_{1}\geq 19 \end{array}\right.\;,\\ n_{2}=\left\{\begin{array}{cc} \left\lceil 21(A_{0}-A_{1})\right\rceil & \mathrm{else}\\ -9 & n_{2}\leq -9 \end{array}\right. \end{array}$$

where ⌊⌋ indicates rounding down to an integer and ⌈⌉ denotes rounding up. Obviously, when $X<\frac{n_{2}}{21}$ or $X\geq \frac{n_{1}}{21}$, f(δ) is constant, thus

(25) $$\begin{array}{c} F_{T0}=\int_{0}^{2\pi }f(\delta )d\tau \\ =\int_{\frac{n_{2}}{21}<X\leq \frac{n_{1}}{21}}f(\delta )d\tau +\int_{\tau_{n2}}^{\tau_{n3}}f(\delta )d\tau +\int_{-\theta }^{\tau_{n1}}f(\delta )d\tau +\int_{\tau_{n4}}^{2\pi -\theta }f(\delta )d\tau \end{array}$$

where

(26) $$\begin{array}{c} \tau_{n1}=\cos^{-1}\left(\frac{n_{1}-21A_{0}}{21A_{1}}\right)-\theta ,\tau_{n2}=\cos^{-1}\left(\frac{n_{2}-21A_{0}}{21A_{1}}\right)-\theta ,\\ \tau_{n3}=2\;\pi -\cos^{-1}\left(\frac{n_{2}-21A_{0}}{21A_{1}}\right),\tau_{n4}=2\;\pi -\cos^{-1}\left(\frac{n_{1}-21A_{0}}{21A_{1}}\right) \end{array}$$

For $\frac{n_{2}}{21}<X\leq \frac{n_{1}}{21}$, the switching plane divides the response into $n_{1}-n_{2}$ intervals. The response curves within each interval are similar, so there is

(27) $$\int_{\frac{n_{2}}{21}<X\leq \frac{n_{1}}{21}}f(\delta )d\tau =\sum_{j=n_{2}+1}^{n_{1}}\int_{\mathrm{Interval}j}f(\delta )d\tau =\sum_{j=n_{2}+1}^{n_{1}}\left(\int_{\tau_{j1}}^{\tau_{j2}}f(\delta )d\tau +\int_{\tau_{j3}}^{\tau_{j4}}f(\delta )d\tau \right)$$

where

(28) $$\begin{array}{c} \tau_{j1}=\cos^{-1}\left(\frac{j-21A_{0}}{21A_{1}}\right)-\theta ,\tau_{j2}=\cos^{-1}\left(\frac{j-1-21A_{0}}{21A_{1}}\right)-\theta ,\\ \tau_{j3}=2\;\pi -\cos^{-1}\left(\frac{j-1-21A_{0}}{21A_{1}}\right)-\theta ,\tau_{j4}=2\;\pi -\cos^{-1}\left(\frac{j-21A_{0}}{21A_{1}}\right)-\theta \end{array}$$

**Figure 10:**
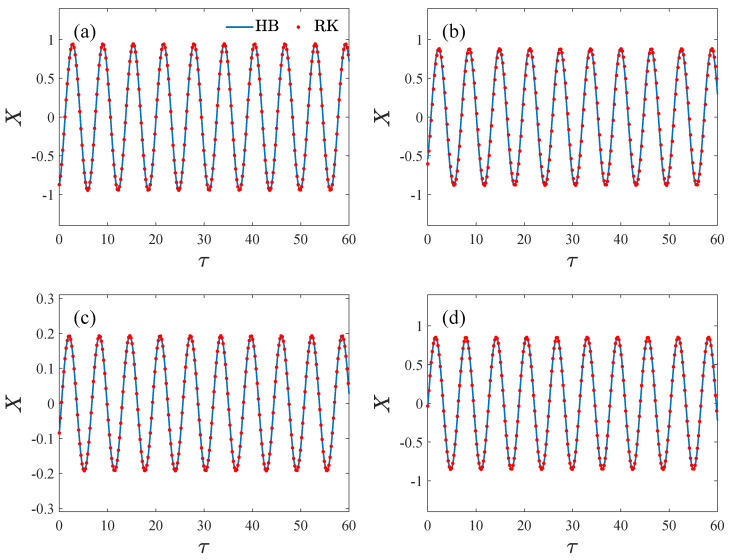
The comparisons between the analytical and numerical solutions, where τ is the non-dimensional time, *X* is the non-dimensional displacement, HB is the analytical solution using the harmonic balance method, and RK denotes the numerical solutions obtained using the Runge–Kutta method. (**a**) $V_{D}=3\;V,V_{A}=20\;\mathrm{mV},V_{T}=3\;V,Q$ = 40,000, ω=18,849.4rad/s, (**b**) $V_{D}=1.75\;V$, $V_{A}=30\;\mathrm{mV},V_{T}=3.75\;V,Q$ = 20,000, ω=18,847rad/s, (**c**) $V_{D}=1\;V,V_{A}=10\;\mathrm{mV},V_{T}=2\;V$, *Q* = 20,000, ω=18,853.7rad/s, (**d**) $V_{D}=4\;V,V_{A}=20\;\mathrm{mV},V_{T}=4\;V,Q=10,000,\omega =18,855\;\mathrm{rad}/s$.

Similarly, $F_{T1}$ and $F_{T2}$ can be obtained. The integral results are given in Appendix B. Figure 10 gives the comparisons between the analytical solutions and the numerical solutions, where τ is the non-dimensional time, *X* is the non-dimensional displacement, HB denotes the solution using the harmonic balance method in this paper, and RK denotes the numerical solutions obtained by the fourth-order Runge–Kutta method. It can be observed that the analytical solutions are in good agreement with the numerical solutions.

The Floquet theory can determine the stability of periodic solutions. The motion equation is rewritten into the state equation as

(29) $$Y^{\prime }=F\left(Y,\tau \right)=\mathrm{AY}+B$$

where

(30) $$F\left(Y,\tau \right)=\left[\begin{array}{c} Y_{2}\\ -\zeta Y_{2}-\Omega^{2}Y_{1}-\alpha {Y_{1}}^{3}+g\left(Y_{1}\right)+c_{4}a_{3}f\left(\delta \right) \end{array}\right]=\mathrm{AY}+\left[\begin{array}{c} 0\\ c_{4}a_{3}f\left(\delta \right) \end{array}\right]$$

$g(Y_{1})$ is the smooth part of the electrostatic force, A is the derivative matrix of the smooth part, and B is the vector of the non-smooth part, where

(31) $$\begin{array}{c} g\left(Y_{1}\right)=F_{D}+F_{S}+\frac{1}{2}c_{4}a_{4}\sum_{i=1}^{N}\frac{1}{{\left(L_{i}-Y_{1}\right)}^{2}}+\frac{1}{2}Nc_{4}a_{4}\frac{1}{{\left(1-Y_{1}\right)}^{2}},\\ A=\left[\begin{array}{cc} 0 & 1\\ -{\Omega^{2}}_{1}-3\alpha {Y_{1}}^{2}+\frac{dg(Y_{1})}{dY_{1}} & -\zeta \end{array}\right],B=\left[\begin{array}{c} 0\\ c_{4}a_{3}f\left(\delta \right) \end{array}\right]. \end{array}$$

**Figure 11:**
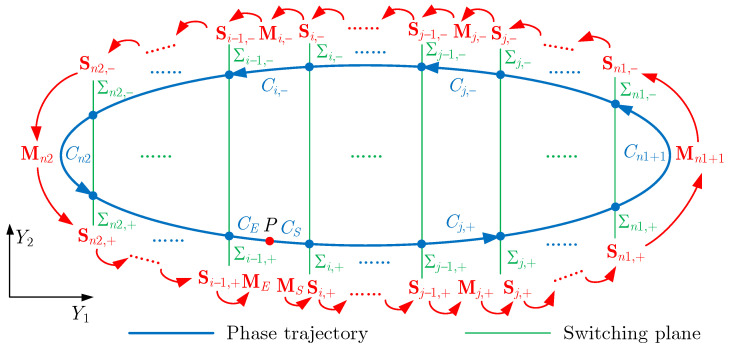
The schematic diagram for calculating the Floquet multiplier matrix of periodic response, where Σ denotes the switching plane, *C* is the section of the phase trajectory, *P* is the starting point (τ=0) and the ending point (τ=2π), and M and S are the corresponding basic solution matrice of the section and switching matrice of the switching plane.

As shown in Figure 11, the phase trajectory of the periodic solution moving counterclockwise is divided into $n_{1}-n_{2}+3$ sections by switching planes Σ and point *P*, where $\Sigma_{j}$ denotes the switching plane at X=j/21 and point *P* corresponds to τ=0 and 2π. We define the section of phase trajectory from the plane $\Sigma_{j-1}$ to $\Sigma_{j}$ as $C_{j,+}$, and the section from the plane $\Sigma_{j}$ to $\Sigma_{j-1}$ as $C_{j,-}$. The plane $\Sigma_{j}$ through which the phase trajectory crosses from left to right is $\Sigma_{j,+}$, and vice versa is $\Sigma_{j,-}$. $\tau_{j,+}$ and $\tau_{j-1,+}$ correspond to the intersection of phase trajectory cross $\Sigma_{j,+}$ and $\Sigma_{j-1,+}$. It is noted that, corresponding to the section of the phase trajectory between two planes, f(δ) is constant and the system is smooth. The system is dis-smooth only on the switching plane f(δ) changes. The Floquet multiplier matrix of periodic response can be determined via the continuous product of basic solution matrices of all smooth sections and switching matrices on the plane as

(32) $$\Phi =M_{E}S_{i-1,+}\cdots S_{j,-}M_{j,-}\cdots S_{j,+}M_{j,+}\cdots S_{i+1,+}M_{i+1}S_{i,+}M_{S}$$

where M and S are the basic solution matrix and switching matrix corresponding to the corresponding section *C* and plane Σ, respectively. $M_{S}$ and $M_{E}$ are the starting and ending basic solution matrices between *P* and the nearest switching plane. The basic solution matrix M is obtained using the following method [22].

To calculate $M_{j,+}$, we divide the non-dimensional time interval $\left[\tau_{j-1,+},\tau_{j,+}\right]$ into $\tilde{N}$ equal parts. The interval is

(33) $$\Delta =\frac{\left(\tau_{j,+}-\tau_{j-1,+}\right)}{\tilde{N}}$$

$C_{\tilde{n}}$ is the approximate matrix of the periodic variable coefficient matrix A in the $\tilde{n}$ th time interval, where

(34) $$C_{\tilde{n}}=A\left(\tilde{\tau }\right),\tilde{\tau }\in \left[\tau_{j-1,+}+\left(\tilde{n}-1\right)\Delta ,\tau_{j-1,+}+\tilde{n}\Delta \right]$$

The basic solution matrix can be given as

(35) $$M_{j,+}=\exp\left(\Delta C_{\tilde{N}}\right)\exp\left(\Delta C_{\tilde{N}-1}\right)\cdots \exp\left(\Delta C_{1}\right)$$

In the same way, we can determine $M_{S},M_{E},M_{j,-}$. The switching matrix S can be obtained using [23]

(36) $$S=I+\frac{\left(F_{+}\left(Y,\tau \right)-F_{-}\left(Y,\tau \right)\right)\cdot n^{T}}{n^{T}\cdot F_{-}\left(Y,\tau \right)}$$

where $n^{T}$ is the normal vector of the switching surface; here, we have $n^{T}={\left[1,0\right]}^{T}$. $F_{+}\left(Y,\tau \right)$ and $F_{-}\left(Y,\tau \right)$ denote the vector fields behind and before the switching plane, respectively. For $\Sigma_{j,+}$, there is

(37) $$\begin{array}{c} F{\left(Y,\tau \right)}_{+}=\left[\begin{array}{c} Y_{2}\\ -\zeta Y_{2}-\Omega^{2}Y_{1}-\alpha {Y_{1}}^{3}+g\left(Y_{1}\right)+c_{4}a_{3}\left(20+2j\right) \end{array}\right],\\ F{\left(Y,\tau \right)}_{-}=\left[\begin{array}{c} Y_{2}\\ -\zeta Y_{2}-\Omega^{2}Y_{1}-\alpha {Y_{1}}^{3}+g\left(Y_{1}\right)+c_{4}a_{3}\left(18+2j\right) \end{array}\right] \end{array}$$

And for $\Sigma_{j,-}$, it is the opposite.

## 4. Bifurcation Analyses of the Resonator System without Tuning

In this section, the singularity theory is used to study the influence of the parameters on the bifurcation behaviors of the untuned resonator. When the tuning voltage equals the DC voltage, $A_{0}$ is very small, so we set $f_{0}=0$ and $A_{0}=0$ in Equations (22) and (23). By defining $A_{1}=u,\theta =v,\omega =\lambda$, Equations (22) and (23) become

(38) $$G_{1}=f_{2}\left(u,v,\lambda \right)=0,G_{2}=f_{3}\left(u,v,\lambda \right)=0$$

The transition sets of Equation (38) are defined as D=B∪H∪DL, where B, H, and DL denote the bifurcation set, the hysteresis set, and the double limit point set [24]. The expressions of the transition sets are given as follows.

Bifurcation set:

(39) $$B=\left\{\left(V_{D},V_{A}\right)\in R^{2}\left|\begin{array}{c} \exists \left(u,v,\lambda \right)\;s.t.\;G_{1}=0,G_{2}=0,G_{1u}G_{2v}-G_{1v}G_{2u}=0,\\ G_{1u}G_{2\lambda }-G_{1\lambda }G_{2u}=0 \end{array}\right.\right\}$$

Hysteresis set:

(40) $$H=\left\{\left(V_{D},V_{A}\right)\in R^{2}\left|\begin{array}{c} \exists \left(u,v,\lambda \right)\;s.t.\;G_{1}=0,G_{2}=0,G_{1u}G_{2v}-G_{1v}G_{2u}=0,\\ G_{1u}\dot{u}-G_{2u}\dot{v}.=0,G_{1u}f_{t2}-G_{2u}f_{t1}=0,\\ f_{t1}=G_{1uu}{\dot{u}}^{2}+2G_{1uv}\dot{u}\dot{v}.+G_{1vv}{\dot{v}}^{2},\\ f_{t2}=G_{2uu}{\dot{u}}^{2}+2G_{2uv}\dot{u}\dot{v}.+G_{2vv}{\dot{v}}^{2} \end{array}\right.\right\}$$

Double limit set:

(41) $$\mathrm{DL}=\left\{\left(V_{D},V_{A}\right)\in R^{2}\left|\begin{array}{c} \exists \left(Z_{1},Z_{2},\lambda \right)\;s.t.\;G_{1}=0,G_{2}=0,G_{1u}G_{2v}-G_{1v}G_{2u}=0,\\ Z=\left(u,v\right),Z_{1}\neq Z_{2} \end{array}\right.\right\}$$

**Figure 12:**
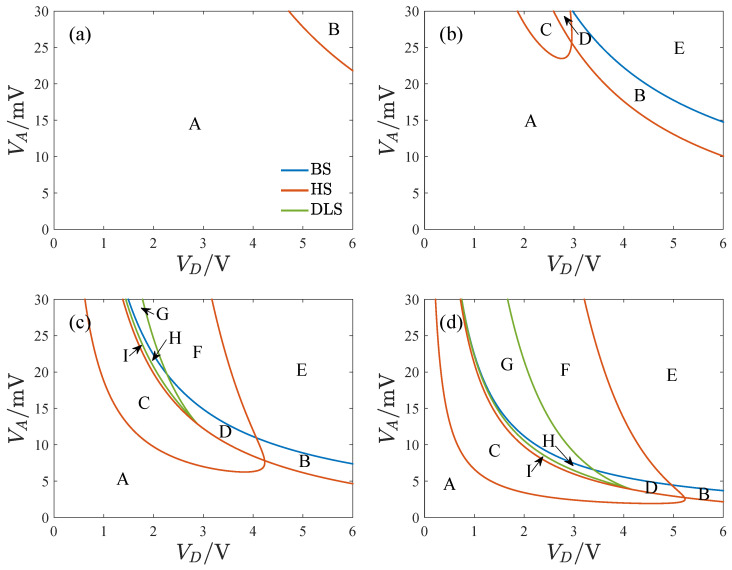
The transition set of Equation (38) on $V_{D}-V_{A}$ plane with different quality factors, where $V_{D}$ is the DC voltage, $V_{A}$ is the amplitude of the AC voltage, Q is the quality factor of the resonator, and BS, HS, and DLS denote the bifurcation set, hysteresis set, and double limit point set, (**a**) Q=5000, (**b**) Q=10,000, (**c**) Q=20,000, (**d**) Q=40,000.

**Figure 13:**
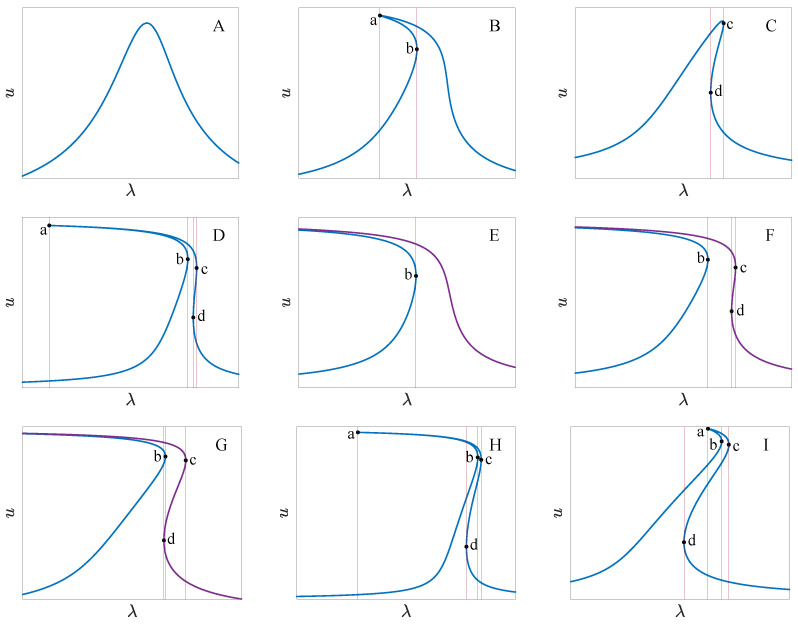
Bifurcation diagrams corresponding to different persist regions of Figure 12, where (**A**–**I**) correspond to regions A through I, respectively.

Figure 12 shows the transition sets on the $V_{D}-V_{A}$ plane with different quality factors where $V_{D}$ is the DC voltage, $V_{A}$ is the amplitude of the AC voltage, Q is the quality factor of the resonator, and BS, HS, and DLS denote the bifurcation set, hysteresis set, and double limit point set. The transition sets dividing the planes into several persistent regions become complicated as the quality factor increases. The number of persistent regions also increases with the quality factor. Figure 13 provides the bifurcation diagrams for different parameter regions. To clarify the topological structure of the bifurcation curves, we have identified four key points (a through d) that mark the turning points of the curves.

The bifurcation diagrams have a single solution branch in all regions except E, F, and G. Region A does not have a turning point, and the bifurcation curve has a single solution for any ω value. Regions B and C have two turning points, with three solutions between the key points. Four key points appear in regions D, H, and I. In region D, point d is to the right of b, and there is a single solution region between points b and d. In region H, point d is to the left of b, resulting in four solutions between b and d. In region I, point d is to the left of a. Regions E, F, and G have bifurcation diagrams with two solution branches. Only one turning point exists in region E. In region F, point d is to point b’s right, while region G is to the left of b. Notably, the curves display hardening in region B, softening in C and E, and a combination of both in regions D and F through I.

**Figure 14:**
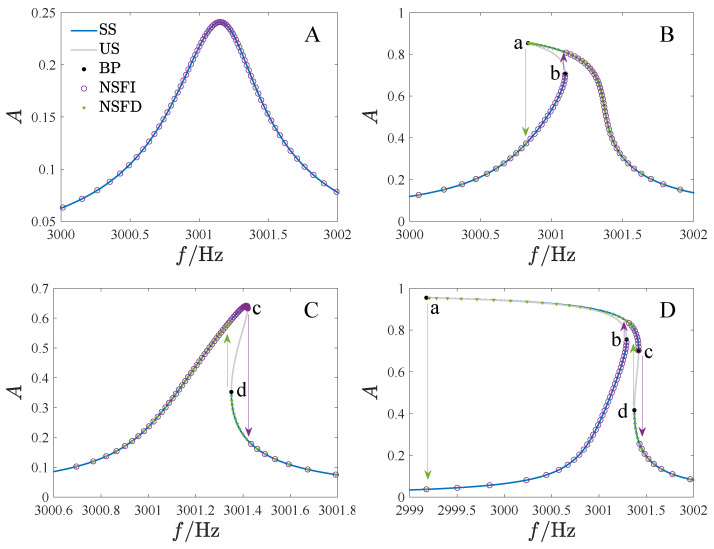

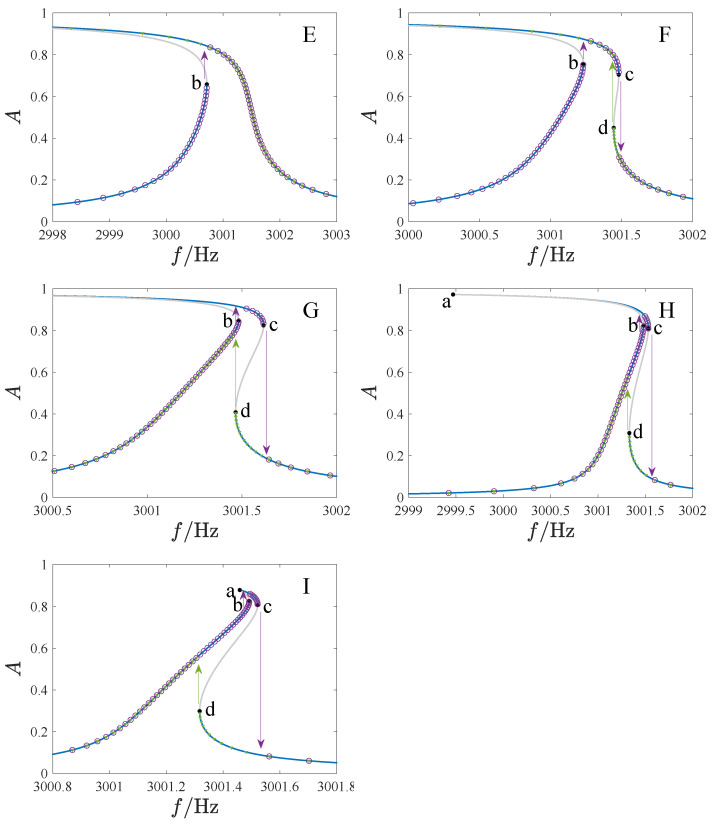
Amplitude–frequency curves corresponding to different regions of Figure 8, where (**A**–**I**) correspond to regions A through I, respectively. SS is the stable analytical solution, US is the unstable analytical solution, BP is the bifurcation point, NSFI denotes the numerical solution as the driving frequency increases, and NSFD denotes the numerical solution as the driving frequency decreases.

In Figure 14, we have provided the frequency–amplitude curves for various regions, where f=ω/2π is the driving frequency, *A* is the non-dimensional amplitude, SS is the stable analytical solution, US is the unstable analytical solution, BP is the bifurcation point, NSFI denotes the numerical solution as the driving frequency increases, and NSFD denotes the numerical solution as the driving frequency decreases. Table 2 contains the corresponding parameter values. Different types of jump phenomena are observed in these regions. Specifically, no jump occurs in region A. In regions B and C, the response jumps once as the frequency increases and decreases. In regions F to I, jump phenomena occur twice with the increase of driving frequency and once with the decrease. In region D, the jump will occur twice when the frequency increases and decreases. However, the response will not jump down in regions E and F when the frequency is reduced. The numerical results presented in Figure 14 align well with the analytical results.

## 5. The Effects of Tuning on the Response of the Resonator

**Figure 15:**
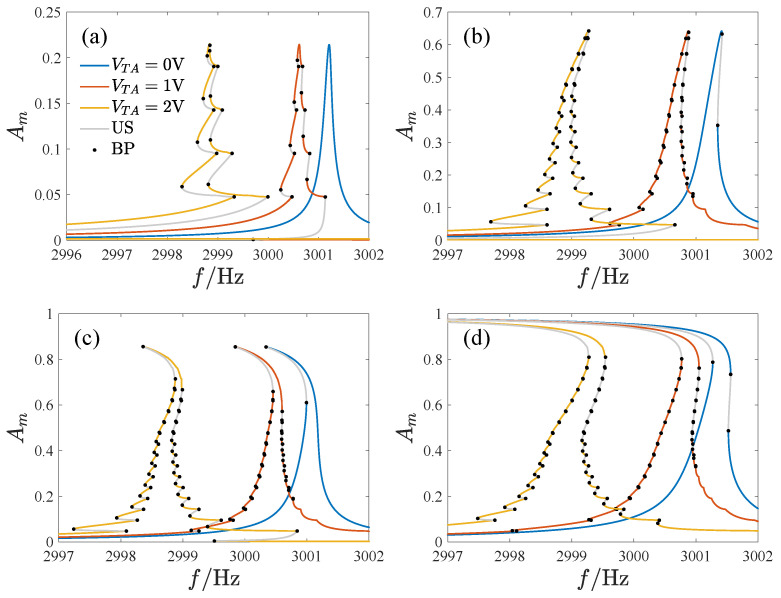
The effects of the tuning voltage on the amplitude–frequency curves of the resonator, where US is the unstable analytical solution, BP is the bifurcation point, (**a**) $V_{D}=1\;V,V_{A}=10\;\mathrm{mV}$, (**b**) $V_{D}=2\;V,V_{A}=15\;\mathrm{mV}$, (**c**) $V_{D}=5\;V,V_{A}=8\;\mathrm{mV}$, and (**d**) $V_{D}=2.6\;V,V_{A}=30\;\mathrm{mV}$.

For this section, the quality factor *Q* is set to 20,000. We identify four groups of DC and AC voltages that correspond to amplitude–frequency curves of the untuned system with different topologies and study the effect of tuning on the response. Figure 15 shows the results of the tuning voltage on the amplitude–frequency curve of the system, where the tuning voltage is defined as $V_{TA}=\left|V_{D}-V_{T}\right|$, and the amplitude $A_{m}=A_{0}+A_{1}$ is the maximum displacement of the response, US is the unstable analytical solution, and BP is the bifurcation point.

**Figure 16:**
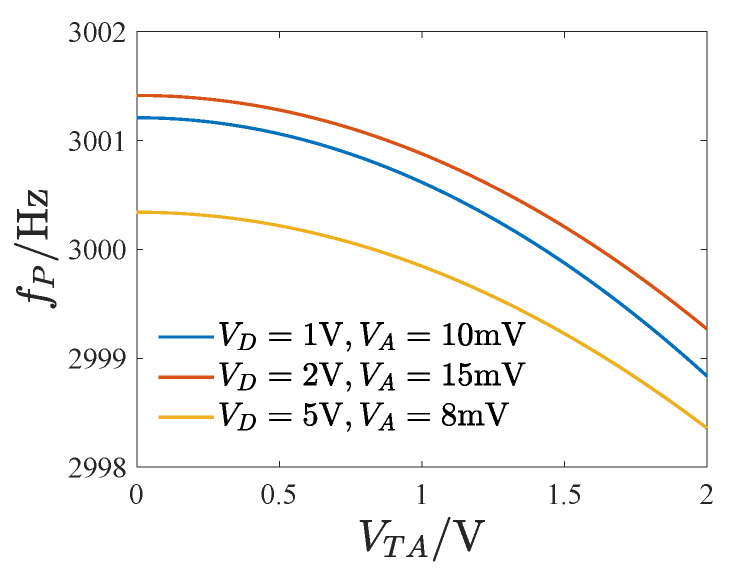
The curve of the peak frequency varying with the tuning voltage.

As the tuning voltage increases, the amplitude–frequency curve shifts leftwards. The amplitude–frequency curves in Figure 15a,c change from non-skewed and softening to a mixture of hardening and softening characteristics, respectively. Additionally, the tuned response curves exhibit many twists, bifurcation points, and unstable solutions, making the amplitude–frequency curve complex. Figure 16 displays the peak frequency curve as the tuning voltage varies, showing that the peak frequency drops faster with a larger tuning voltage, where $f_{P}$ denotes the peak frequency of the amplitude–frequency curve.

**Figure 17:**
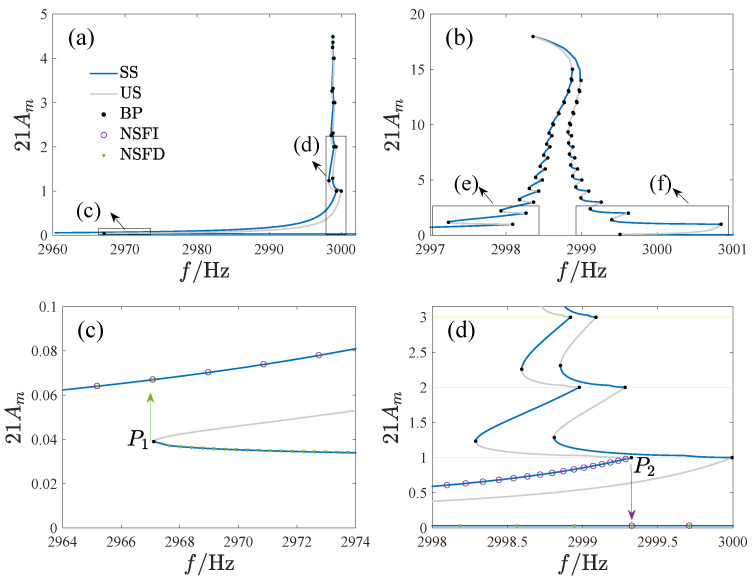

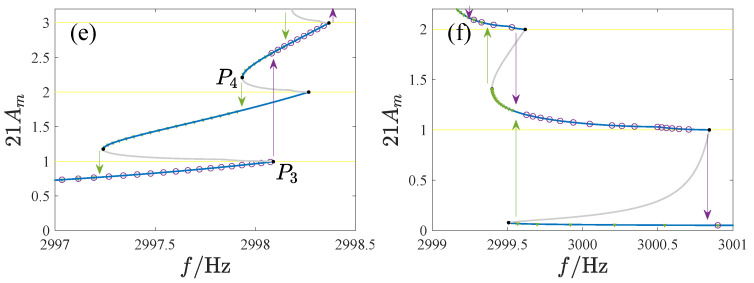
The amplitude–frequency curves and those of the resonator under tuning. SS is the stable analytical solution, US is the unstable analytical solution, BP is the bifurcation point, NSFI denotes the numerical solution as driving frequency is increased, NSFD denotes the numerical solution as driving frequency is decreased, and the yellow lines denote the switching planes. (**a**) $V_{D}=1\;V,V_{A}=10\;\mathrm{mV}$, (**b**) $V_{D}=5\;V,V_{A}=8\;\mathrm{mV}$, (**c**,**d**) are the local enlargements of (**a**), (**e**,**f**) are the local enlargements of (**b**).

Figure 17 gives the amplitude–frequency curve and its local enlargement, with the ordinate magnified 21 times, where SS is the stable analytical solution, US is the unstable analytical solution, BP is the bifurcation point, NSFI denotes the numerical solution as driving frequency is increased, NSFD denotes the numerical solution as driving frequency is decreased, and the yellow lines denote the switching planes. The numerical results confirm the analytical solution. Two types of bifurcation points exist on the curve: smooth ($P_{1}$ in Figure 17c and $P_{4}$ in Figure 17e) and dis-smooth ($P_{2}$ in Figure 17d and $P_{3}$ in Figure 17e). The amplitude of all dis-smooth bifurcation points is an integer multiple of 1/21, indicating that they occur on the switching plane of non-uniform fingers. As the frequency changes, the system response undergoes complicated jumps. In Figure 17c,d, the response jumps down at point $P_{1}$ as the frequency increases and jumps up at point $P_{2}$ when the frequency decreases. In Figure 17e, the response does not reach the nearest solution after jumping up at point $P_{3}$ as the frequency increases but rather jumps to a solution with a larger amplitude.

Considering that there is a stable solution and an unstable solution between every two switching planes, this provided the possibility to achieve the desired periodic response between the switching planes by designing the length of the fingers reasonably, and quickly switching between multiple periodic responses by jumping. However, operating the tuning resonators in the open-loop mode is not advisable, as the response can easily jump to other solutions when disturbed, resulting in an incorrect output.

## 6. Conclusions

This paper studies the nonlinear vibration response of a MEMS resonator with a triangular tuning comb. According to Newton’s second law, the dynamic equation of the resonator is derived, and it is found that the tuned electrostatic force of the triangular tuning comb is dis-smooth. The harmonic balance and section integral methods are used to obtain the analytical solution of the dis-smooth system. The stability of the periodic response was studied based on the Floquet theory. The numerical results show an excellent agreement with the analytical solutions.

The singularity theory is used to investigate the bifurcation of the periodic response of the untuned system. The transition sets on the DC-AC voltage plane corresponding to different quality factors are obtained, which divide the planes into several persistent regions. The topological structures of the bifurcation diagrams corresponding to different parameter regions are analyzed. Abundant jump phenomena of the periodic response, varying with the driving frequency, are present.

The effects of tuning voltage on the system response are studied. We demonstrated that, with the increase of the tuning voltage, the amplitude–frequency curve moves to the left and presents more hardening characteristics. Many twists, bifurcation points, and unstable solutions appear on the curve, which leads to complicated jump phenomena. Two bifurcation points exist, the smooth bifurcation points and the dis-smooth bifurcation points, where the dis-smooth bifurcation points occur on the switching plane of the non-uniform comb.

## Figures and Tables

**Table 1:** Parameters of the MEMS resonator [1].

Parameters	Values
Proof mass, *m*	$1.3665\times 10^{-9}$ kg
Length of the supporting beam, *L*	420 μm
Width of the supporting beam, *b*	2 μm
Structure thickness, *h*	30 μm
Young’s modulus, *E*	150 GPa
Finger width, *w*	3 μm
Finger spacing, *d*	1.5 μm
Dielectric constant, ε	$8.85\times 10^{-12}$ F/m
Number of fingers, *N*	58
Length of the uniform finger, $l_{b}$	60 μm
Static overlap of the uniform finger, *l*	38 μm
Static lateral separation of the uniform finger, $x_{0}$	42 μm
Length of the uniform finger, $l_{i}$	4 to 60 μm, step size 2 μm

**Table 2:** Values of parameters corresponding to Figure 14A–I.

Figures	$V_{D}$/V	$V_{A}$/mV	*Q*
14A	3	15	5000
14B	4	20	10,000
14C	2	15	20,000
14D	3	14.6	20,000
14E	5	30	10,000
14F	3	20	40,000
14G	1.75	30	20,000
14H	2	11.1	40,000
14I	2	10.3	40,000

## Data Availability

The data that support the findings of this study are available from the corresponding author (Lijuan Zhang) upon reasonable request.

## Appendix A

We take $F_{D0}$ as an example to illustrate the solution process. For simplicity, the solution can be written as

(A1) $$X=A_{0}+A_{11}\cos\tau +A_{12}\sin\tau$$

where

(A2) $$A_{11}=A_{1}\cos\theta ,A_{12}=-A_{1}\sin\theta$$

By setting z=exp(iψ), we have

(A3) $$\cos\psi =\frac{z^{2}+1}{2z},\sin\psi =\frac{z^{2}-1}{2iz}$$

where $i=\sqrt{-1}$. By substituting (A1) into $F_{D0}$, and according to the residue theorem,

(A4) $$\begin{array}{c} F_{D0}=\int_{0}^{2\pi }F_{D}d\tau =\int_{0}^{2\pi }C{\left(\beta \cos\tau -1\right)}^{2}\left(-a_{1}-\frac{a_{2}}{{\left(1-X\right)}^{2}}\right)d\tau \\ =\oint_{\left|z\right|=1}f_{D}\left(z\right)dz=-2\pi i\sum \mathrm{Res}[f_{D}\left(z\right),z_{k}] \end{array}$$

where

(A5) $$f_{D}\left(z\right)=\frac{i{\left(z^{2}\beta +\beta -2z\right)}^{2}\left(\begin{array}{c} {A_{11}}^{2}a_{1}z^{4}-{A_{12}}^{2}a_{1}z^{4}+4A_{0}A_{11}\;a_{1}\;z^{3}+4iA_{12}a_{1}z\;\\ +4a_{1}\;z^{2}{A_{0}}^{2}+4\;iA_{0}A_{12}\;a_{1}\;z+2{A_{11}}^{2}a_{1}z^{2}+4A_{11}a_{1}z^{3}\;\\ +2{A_{12}}^{2}a_{1}z^{2}+4A_{0}A_{11}a_{1}z+8A_{0}a_{1}z^{2}+2\;iA_{11}A_{12}a_{1}\;\\ -4iA_{12}a_{1}z^{3}+{A_{11}}^{2}a_{1}+4A_{11}a_{1}z-{A_{12}}^{2}a_{1}\;\\ +4a_{1}\;z^{2}+4a_{2}\;z^{2}-2\;iA_{11}A_{12}\;a_{1}\;z^{4}-4\;iA_{0}A_{12}\;a_{1}\;z^{3} \end{array}\right)}{4\;z^{3}{\left(-iA_{12}\;z^{2}+A_{11}\;z^{2}+2\;A_{0}z+iA_{12}+A_{11}+2\;z\right)}^{2}}$$

$z_{k}$ is the isolated singularity of $f_{D}\left(z\right)$ contained in the unit circle. There exist two isolated singularities, as shown below.

(A6) $$z_{1}=0,z_{2}=-\frac{A_{0}+1-\sqrt{{A_{0}}^{2}-{A_{11}}^{2}-{A_{12}}^{2}+2A_{0}+1}}{-iA_{12}+A_{11}}$$

where $z_{1}$ and $z_{2}$ are poles of order three and order two, respectively. Then,

(A7) $$\mathrm{Res}\left[f_{D}\left(z\right),z_{k}\right]=\lim_{z\to z_{1}}\frac{d^{2}}{dz^{2}}\left\{{\left(z-z_{1}\right)}^{3}f_{D}\left(z\right)\right\}+\lim_{z\to z_{2}}\frac{d}{dz}\left\{{\left(z-z_{2}\right)}^{2}f_{D}\left(z\right)\right\}$$

Substituting (A7) and (A2) into (A4) results in

(A8) $$\begin{array}{c} F_{D0}=C[\left(2\;{A_{0}}^{3}-3\;A_{0}{A_{1}}^{2}+6\;{A_{0}}^{2}-3\;{A_{1}}^{2}+6\;A_{0}+2\right)f_{A1}\beta^{2}a_{2}\cos2\;\theta \;\\ +\beta^{2}a_{2}\left(4\;{A_{0}}^{2}{A_{1}}^{2}-2\;{A_{0}}^{4}-2\;{A_{1}}^{4}-8\;{A_{0}}^{3}+8\;A_{0}{A_{1}}^{2}-12\;{A_{0}}^{2}+4\;{A_{1}}^{2}-8\;A_{0}-2\right)\cos2\;\theta \;\\ -4\beta \;{A_{1}}^{3}a_{2}f_{A1}\cos\theta +{A_{1}}^{2}(-{A_{0}}^{4}+2\;{A_{0}}^{2}{A_{1}}^{2}-{A_{1}}^{4}-4\;{A_{0}}^{3}+4\;A_{0}{A_{1}}^{2}-6\;{A_{0}}^{2}\;\\ +2\;{A_{1}}^{2}-4\;A_{0}-1)\beta^{2}a_{1}-{A_{1}}^{2}\left(A_{0}+1\right)\beta^{2}a_{2}f_{A1}+{A_{1}}^{2}(-2{A_{0}}^{4}+4{A_{0}}^{2}{A_{1}}^{2}-2{A_{1}}^{4}\;\\ -8\;{A_{0}}^{3}+8\;A_{0}{A_{1}}^{2}-12\;{A_{0}}^{2}+4\;{A_{1}}^{2}-8\;A_{0}-2)a_{1}-2{A_{1}}^{2}\left(A_{0}+1\right)a_{2}f_{A1}]\;\\ /[2\;{A_{1}}^{2}{\left(A_{0}+1+A_{1}\right)}^{2}{\left(A_{0}+1-A_{1}\right)}^{2}] \end{array}$$

Similarly, we obtain

(A9) $$\begin{array}{c} F_{D1}=C[f_{A1}\beta a_{2}\left(-8{A_{0}}^{3}A_{1}+12A_{0}{A_{1}}^{3}-24{A_{0}}^{2}A_{1}+12{A_{1}}^{3}-24A_{0}A_{1}-8A_{1}\right)\cos2\theta \;\\ +f_{A1}a_{2}(8{A_{0}}^{4}A_{1}-16{A_{0}}^{2}{A_{1}}^{3}+8{A_{1}}^{5}+32{A_{0}}^{3}A_{1}-32A_{0}{A_{1}}^{3}+48{A_{0}}^{2}A_{1}-16{A_{1}}^{3}+32A_{0}A_{1}\;\\ +8A_{1})\cos2\theta +\left(3\beta^{2}+4\right){A_{1}}^{4}f_{A1}a_{2}\cos\theta +(-8{A_{0}}^{4}+12{A_{0}}^{2}{A_{1}}^{2}-3{A_{1}}^{4}-32{A_{0}}^{3}+24A_{0}{A_{1}}^{2}\;\\ -48{A_{0}}^{2}+12{A_{1}}^{2}-32A_{0}-8)f_{A1}a_{2}\beta^{2}\cos3\theta ]/\left[4{A_{1}}^{2}{\left(A_{0}+1+A_{1}\right)}^{2}{\left(A_{0}+1-A_{1}\right)}^{2}\right] \end{array}$$

(A10) $$\begin{array}{c} F_{D2}=C[(8\;{A_{0}}^{4}-12\;{A_{0}}^{2}{A_{1}}^{2}+3\;{A_{1}}^{4}+32\;{A_{0}}^{3}-24\;A_{0}{A_{1}}^{2}+48\;{A_{0}}^{2}-12\;{A_{1}}^{2}+32\;A_{0}\;\\ +8)a_{2}f_{A1}\beta^{2}\sin\left(3\;\theta \right)+(-8\;{A_{0}}^{5}+16\;{A_{0}}^{3}{A_{1}}^{2}-8\;A_{0}{A_{1}}^{4}-40\;{A_{0}}^{4}+48\;{A_{0}}^{2}{A_{1}}^{2}-8\;{A_{1}}^{4}\;\\ -80\;{A_{0}}^{3}+48\;A_{0}{A_{1}}^{2}-80\;{A_{0}}^{2}+16\;{A_{1}}^{2}-40\;A_{0}-8)a_{2}\beta^{2}\sin\left(3\;\theta \right)+(8\;{A_{0}}^{3}A_{1}\;\\ -12\;A_{0}{A_{1}}^{3}+24\;{A_{0}}^{2}A_{1}-12\;{A_{1}}^{3}+24\;A_{0}A_{1}+8\;A_{1})a_{2}\sin\left(2\;\theta \right)\beta \;f_{A1}+(-8\;{A_{0}}^{4}A_{1}\;\\ +16\;{A_{0}}^{2}{A_{1}}^{3}-8\;{A_{1}}^{5}-32\;{A_{0}}^{3}A_{1}+32\;A_{0}{A_{1}}^{3}-48\;{A_{0}}^{2}A_{1}+16\;{A_{1}}^{3}-32\;A_{0}A_{1}\;\\ -8\;A_{1})a_{2}\sin\left(2\;\theta \right)\beta -{A_{1}}^{4}a_{2}f_{A1}\left(\beta^{2}+4\;\right)\sin\left(\theta \right)]/\left[4{A_{1}}^{3}{\left(A_{0}+1+A_{1}\right)}^{2}{\left(A_{0}+1-A_{1}\right)}^{2}\right] \end{array}$$

(A11) $$\begin{array}{c} F_{S0}=C[\left({A_{0}}^{4}-2\;{A_{0}}^{2}{A_{1}}^{2}+{A_{1}}^{4}-4\;{A_{0}}^{3}+4\;A_{0}{A_{1}}^{2}+6\;{A_{0}}^{2}-2\;{A_{1}}^{2}-4\;A_{0}+1\right)a_{1}\\ +\left(1-A_{0}\right)a_{2}f_{A2}]/\left[{\left(A_{0}-1+A_{1}\right)}^{2}{\left(A_{0}-1-A_{1}\right)}^{2}\right] \end{array}$$

(A12) $$F_{S1}=Ca_{2}f_{A2}A_{1}\cos\theta /\left[{\left(A_{0}-1+A_{1}\right)}^{2}{\left(A_{0}-1-A_{1}\right)}^{2}\right]$$

(A13) $$F_{S2}=-Cf_{A2}A_{1}a_{2}\sin\theta /\left[{\left(A_{0}-1+A_{1}\right)}^{2}{\left(A_{0}-1-A_{1}\right)}^{2}\right]$$

(A14) $$F_{T01}=\frac{L_{i}-A_{0}}{{\left(A_{0}-A_{1}-L_{i}\right)}^{3/2}{\left(A_{0}+A_{1}-L_{i}\right)}^{3/2}}$$

(A15) $$F_{T02}=\frac{1-A_{0}}{{\left(A_{0}-A_{1}-1\right)}^{3/2}{\left(A_{0}+A_{1}-1\right)}^{3/2}}$$

(A16) $$F_{T11}=\frac{A_{1}\cos\theta }{{\left(A_{0}-A_{1}-L_{i}\right)}^{3/2}{\left(A_{0}+A_{1}-L_{i}\right)}^{3/2}}$$

(A17) $$F_{T12}=\frac{A_{1}\cos\theta }{{\left(A_{0}-A_{1}-1\right)}^{3/2}{\left(A_{0}+A_{1}-1\right)}^{3/2}}$$

(A18) $$F_{T21}=\frac{-A_{1}\sin\theta }{{\left(A_{0}-A_{1}-L_{i}\right)}^{3/2}{\left(A_{0}+A_{1}-L_{i}\right)}^{3/2}}$$

(A19) $$F_{T22}=\frac{-A_{1}\sin\theta }{{\left(A_{0}-A_{1}-1\right)}^{3/2}{\left(A_{0}+A_{1}-1\right)}^{3/2}}$$

(A20) $$F_{T0}=\frac{A_{0}}{2}\left(2\;{A_{0}}^{2}\alpha +3\;{A_{1}}^{2}\alpha +2\;{\Omega_{0}}^{2}\right)$$

(A21) $$F_{T1}=\frac{A_{1}}{8}\left[\left(12\;{A_{0}}^{2}\alpha +3\;\alpha \;{A_{1}}^{2}+4\;{\Omega_{0}}^{2}-4\;\right)\cos\theta -4\zeta \sin\theta \right]$$

(A22) $$F_{T2}=-\frac{A_{1}}{8}\left[\left(12\;\alpha \;{A_{0}}^{2}+3\;\alpha \;{A_{1}}^{2}+4{\Omega_{0}}^{2}-4\;\right)\sin\theta +4\zeta \cos\theta \right]$$

where

(A23) $$f_{A1}=\sqrt{{A_{0}}^{2}-{A_{1}}^{2}+2\;A_{0}+1},f_{A2}=\sqrt{{A_{0}}^{2}-{A_{1}}^{2}-2\;A_{0}+1}$$

## Appendix B

The integral results are given as follows:

(A24) $$\begin{array}{c} \int_{\tau_{j1}}^{\tau_{j2}}f(\delta )d\tau +\int_{\tau_{j3}}^{\tau_{j4}}f(\delta )d\tau \;\\ =4\;j\left(\cos^{-1}\left(\;\frac{21A_{0}-j}{21A_{1}}\right)-\cos^{-1}\left(\;\frac{1+21A_{0}-j}{21A_{1}}\right)\right)-36\cos^{-1}\left(\;\frac{1+21A_{0}-j}{21A_{1}}\right)+36\cos^{-1}\left(\frac{21A_{0}-j}{21A_{1}}\right) \end{array}$$

(A25) $$\begin{array}{c} \int_{\tau_{j1}}^{\tau_{j2}}f(\delta )\cos\tau d\tau +\int_{\tau_{j3}}^{\tau_{j4}}f(\delta )\cos\tau d\tau \;\\ =\frac{4\;}{21}\cos\theta \left(\begin{array}{c} \sqrt{\frac{441{A_{1}}^{2}-j^{2}+42jA_{0}-441{A_{0}}^{2}+2j-42A_{0}-1}{{A_{1}}^{2}}}j-\sqrt{\frac{441A^{2}-j^{2}+42jA_{0}-441{A_{0}}^{2}}{{A_{1}}^{2}}}j\\ +9\;\sqrt{\frac{441{A_{1}}^{2}-j^{2}+42jA_{0}-441{A_{0}}^{2}+2j-42A_{0}-1}{A^{2}}}-9\;\sqrt{\frac{441{A_{1}}^{2}-j^{2}+42jA_{0}-441{A_{0}}^{2}}{{A_{1}}^{2}}} \end{array}\right) \end{array}$$

(A26) $$\begin{array}{c} \int_{\tau_{j1}}^{\tau_{j2}}f(\delta )\sin\tau d\tau +\int_{\tau_{j3}}^{\tau_{j4}}f(\delta )\sin\tau d\tau \\ =-\frac{4\;}{21}\sin\theta \left(\begin{array}{c} \sqrt{\frac{44{A_{1}}^{2}-j^{2}+42jA_{0}-441{A_{0}}^{2}+2j-42A_{0}-1}{{A_{1}}^{2}}}j-\sqrt{\frac{441{A_{1}}^{2}-j^{2}+42jA_{0}-441{A_{0}}^{2}}{{A_{1}}^{2}}}j\\ +9\;\sqrt{\frac{441{A_{1}}^{2}-j^{2}+42jA_{0}-441{A_{0}}^{2}+2j-42A_{0}-1}{{A_{1}}^{2}}}-9\;\sqrt{\frac{441{A_{1}}^{2}-j^{2}+42jA_{0}-441{A_{0}}^{2}}{{A_{1}}^{2}}} \end{array}\right) \end{array}$$

(A27) $$\int_{-\theta }^{\tau_{n1}}f(\delta )d\tau +\int_{\tau_{n4}}^{2\pi -\theta }f(\delta )d\tau =4\;n_{1}\cos^{-1}\left(\frac{\;n_{1}-21A_{0}}{21A_{1}}\right)+40\;\cos^{-1}\left(\frac{\;n_{1}-21A_{0}}{21A_{1}}\right)$$

(A28) $$\int_{-\theta }^{\tau_{n1}}f(\delta )\cos\tau d\tau +\int_{\tau_{n4}}^{2\pi -\theta }f(\delta )\cos\tau d\tau =\frac{4\;\left(n_{1}+10\right)\cos\theta \;}{21}\sqrt{\frac{441\;{A_{1}}^{2}-441\;{A_{0}}^{2}+42\;A_{0}n_{1}-{n_{1}}^{2}}{{A_{1}}^{2}}}$$

(A29) $$\int_{-\theta }^{\tau_{n1}}f(\delta )\sin\tau d\tau +\int_{\tau_{n4}}^{2\pi -\theta }f(\delta )\sin\tau d\tau =-\frac{4\;\left(n_{1}+10\right)\sin\theta \;}{21}\sqrt{\frac{441\;{A_{1}}^{2}-441\;{A_{0}}^{2}+42\;A_{0}n_{1}-{n_{1}}^{2}}{{A_{1}}^{2}}}$$

(A30) $$\int_{\tau_{n2}}^{\tau_{n3}}f(\delta )d\tau =4\;\cos^{-1}\left(\frac{21\;A_{0}-n_{2}}{21A_{1}}\right)\left(n_{2}+9\right)$$

(A31) $$\int_{\tau_{n2}}^{\tau_{n3}}f(\delta )\cos\tau d\tau =-\frac{4\;\left(n_{2}+9\right)\cos\theta }{21}\sqrt{\frac{441\;{A_{1}}^{2}-441\;{A_{0}}^{2}+42\;A_{0}n_{2}-{n_{2}}^{2}}{{A_{1}}^{2}}}$$

(A32) $$\int_{\tau_{n2}}^{\tau_{n3}}f(\delta )\sin\tau d\tau =\frac{4\;\left(n_{2}+9\right)\sin\theta \;}{21}\sqrt{\frac{441\;{A_{1}}^{2}-441\;{A_{0}}^{2}+42\;A_{0}n_{2}-{n_{2}}^{2}}{{A_{1}}^{2}}}$$
